# The clinical utility of thermal ablation procedures in thyroid nodules: Latin American Thyroid Society (LATS) surgical affairs committee expert opinion. Part 2

**DOI:** 10.20945/2359-4292-2025-0129

**Published:** 2025-11-22

**Authors:** Juan Pablo Dueñas, Erivelto Martinho Volpi, Ana Voogd, Álvaro Sanabria, Santiago Zund, José Luis Novelli, Luiz Paulo Kowalski

**Affiliations:** 1 División de Cirugía Endocrina, Clínica de Cirugía Endocrina Integral, Torre Médica Tesoro 2, Medellín, Colombia; 2 Departamento de Cirurgia de Cabeça e Pescoço, Hospital Alemão Oswaldo Cruz, SP, Brasil; 3 Departamento de Cirugía de Cabeza y Cuello, Hospital Universitario Austral, Buenos Aires, Argentina; 4 Departamento de Cirugía, Facultad de Medicina, Universidad de Antioquia, CEXCA, Centro de Excelencia en Enfermedades de Cabeza y Cuello, Medellín, Colombia; 5 Departamento de Cirugía de Cabeza y Cuello, Instituto de Oncología A. Roffo, Buenos Aires, Argentina; 6 Departamento de Cirugía de Cabeza y Cuello, Centro de Tiroides de Rosario, Argentina; 7 Departamento de Cirurgia de Cabeça e Pescoço e Otorrinolaringologia, A.C. Camargo Cancer Center, São Paulo, SP, Brasil

**Keywords:** Thyroid nodule, ablation techniques, radiofrequency ablation, laser therapy, hyperthyroidism, thyroid cancer, papillary

## Abstract

Thermal ablation (TA) encompasses various options such as radiofrequency ablation (RFA), microwave ablation (MWA), laser ablation (LA), and high-intensity focused ultrasound (HIFU). The fundamental principle of these techniques involves generating heat to induce coagulative necrosis of the nodules. The rising incidence of thyroid nodules, most of which are benign, has highlighted the importance of minimally invasive methods that effectively control symptoms, address cosmetic concerns, and achieve volume reduction. The potential complications associated with surgical interventions have driven the widespread adoption of TA modalities, now used not only for symptomatic benign thyroid nodules (BTN), including autonomously functioning thyroid nodules (AFTN), but also for low-risk papillary thyroid microcarcinoma (PTMC). The evidence presented in this consensus has demonstrated the comparable effectiveness of TA to surgery for BTN in terms of volume reduction percentage (VRP), resolution of symptoms, and cosmetic concerns. Similarly, TA could be considered a suitable option for treating AFTN when surgery or radioactive iodine (RAI) is contraindicated, or when patients decline either of these options, offering a comparable effectiveness profile to RAI in terms of normalizing thyroid-stimulating hormone levels. For PTMC, TA may serve as an alternative for patients at high surgical risk or those who decline surgery, showing comparable outcomes to surgery in terms of local recurrence and lymph node metastasis. Additionally, TA exhibits a superior safety profile compared to surgery or RAI, characterized by reduced complications, preservation of thyroid function, and shorter hospitalization durations. While evidence on cost-effectiveness in Latin America remains limited, studies conducted in other countries support the implementation of TA as a first-line treatment option for BTN. The lack of economic assessment specific to AFTN complicates its consideration as a primary treatment choice; however, the effectiveness and safety profile suggest that the widespread adoption of TA as a first-line therapy could be considered for carefully selected patients diagnosed with AFTN or PTMC. The Surgical Affairs Committee of the Latin American Thyroid Society conducted a comprehensive review of TA as a primary treatment modality for benign, autonomously functioning, and malignant thyroid nodules to ensure its appropriate utilization in the field.

## INTRODUCTION

In an era characterized by rapid technological advancement, there is a global increase in the incidence of thyroid nodules, regardless of a country’s development level or economic status. This trend is largely attributed to the widespread use of highly sensitive imaging techniques (^1^). Consequently, there is growing recognition of the urgent need to explore alternative therapeutic interventions beyond surgery, which is currently overutilized in the management of thyroid nodules. Surgical procedures necessitate hormonal replacement therapy and carry potential risks of complications such as vocal cord palsy, hypocalcemia, infection, hematoma, or hemorrhage (^2^). Even with lobectomy, the rates of thyroid hormone supplementation can reach up to 59.3% for thyroid cancer and 39.4% for benign nodules (^3^). This underscores the importance of pursuing safer treatment options.

Thermal ablation (TA) encompasses several techniques, including radiofrequency ablation (RFA), microwave ablation (MWA), laser ablation (LA), and high-intensity focused ultrasound (HIFU). The fundamental principle of TA is generating heat through an electrode needle in RFA, an antenna in MWA, or a fiber in LA to induce coagulative necrosis of the nodules (^4^). Bibliometric studies have shown that the number of published articles has dramatically increased from 2 in 2000 to 140 in 2021, indicating a growing trend in the application of minimally invasive procedures (^5^), not only for benign nodules but also for low-risk papillary thyroid microcarcinoma (PTMC) (^6^).

The primary objective of currently available thermal therapies is to provide a procedure that, compared to surgery, results in fewer and usually transient side effects; can be performed on an outpatient basis; is less expensive and time-consuming; and preserves the remaining portion of the gland, leading to a lower incidence of induced hypothyroidism. This results in an improvement in health-related quality of life (^7^).

Currently, treatment approaches and therapeutic protocols are shifting toward a patient-centered focus. This approach has prompted the Surgical Affairs Committee of the Latin American Thyroid Society to develop this consensus document to evaluate the efficacy, safety, and feasibility of implementing new technologies for treating benign, autonomously functioning, and selected malignant thyroid nodules. This consensus has been divided into two parts for this purpose. This second part examines TA as a primary treatment strategy for benign, autonomously functioning, and malignant thyroid nodules.

### Benign, non-functioning thyroid nodules

Benign thyroid nodules represent 85%-95% of all thyroid nodules (^5^). A considerable number of patients experience pressure symptoms and express cosmetic concerns, which may compel them to seek surgical interventions despite the nodules being benign. Often, these patients are not presented with non-surgical treatment options such as TA (^8^). The selection of a TA technique is generally influenced by the availability of resources and the treating physician’s expertise (^9^). Among these techniques, RFA has demonstrated a significantly greater reduction in nodule volume compared to LA (^10^) and MWA (^11^). Numerous professional societies have issued guidelines to determine appropriate candidates for TA (8,12-19). These guidelines consistently recommend TA as the primary treatment option for non-functioning benign thyroid nodules in patients who have symptoms or cosmetic concerns, prefer not to undergo surgery, or have nodules larger than 2 cm that show evidence of growth during the follow-up period (^20^,^21^). Notably, Guo and cols. (^22^) conducted a study on multinodular goiter, incorporating various guidelines for thermal ablation. According to their findings, the indications for RFA include having 2-5 nodules with at least 50% normal tissue remaining. These nodules should appear benign on ultrasound, be confirmed as Bethesda class II on cytology, have a diameter of ≥ 20 mm, and/or cause cosmetic or symptomatic issues. This underscores the necessity of individually monitoring and treating each nodule within the context of multinodular goiter (^23^).

### Literature research

A comprehensive search of the MEDLINE database was conducted to identify original studies on the efficacy and safety of thermal ablation for treating benign non-functioning thyroid nodules. The search used the following terms: (((“Minimally invasive therapy”) OR (“laser ablation”) OR (“radiofrequency ablation”) OR (“microwave ablation”) OR (“thermal ablation”)) AND ((“thyroid nodules”) OR (“benign thyroid nodule”) OR (“thyroid nodule”))). The search was not limited by date and continued until April 1, 2024.

### Inclusion criteria

Included studies were: (a) Meta-analyses or systematic reviews of randomized controlled trials (RCTs) or observational studies on patients with benign thyroid nodules treated with thermal ablation, and (b) those that reported follow-up results regarding efficacy or safety.

### Effectiveness

**Figure 1:**
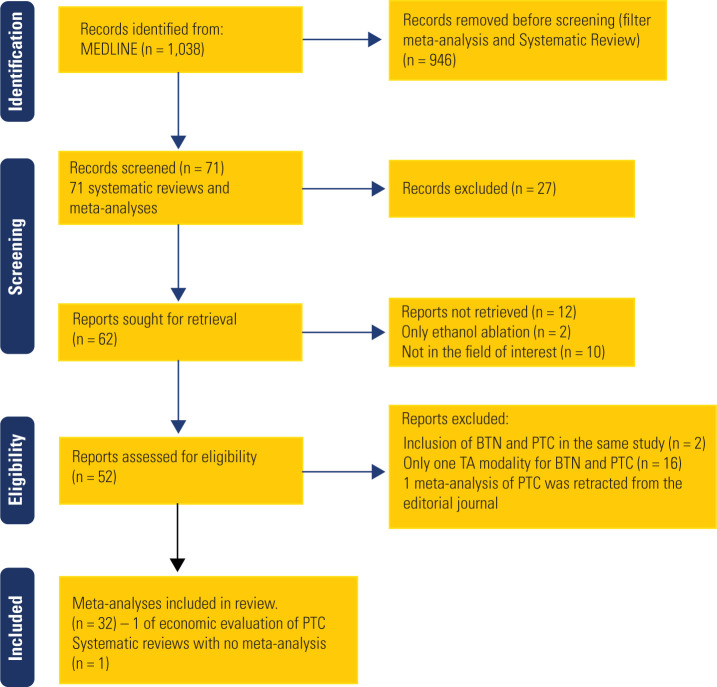
PRISMA Flow Diagram.

Abbreviations: BTN: benign thyroid nodules; PTC: papillary thyroid cancer.

Twenty-four systematic reviews assessed the effectiveness of various treatments, and we included 13 in our analysis (**Figure 1** and **Table 1**) (^24^-^36^). The variables used for this assessment included:

**Table 1:** Effectiveness of thermal ablation in benign thyroid nodules

Study/period	Number and type of studies	Initial nodular volume	TA employed # patients	Effectiveness/improvement			Follow-up
				VRP	Symptoms	Aesthetics	
Guo and cols. (^24^) 2016 -2019	5 cohort studies	1-60 mL	RFA: 899 patients with 956 BTN MWA: 869 patients with 938 BTN	3 m: RFA: -56%. MWA: -54% 6 m: RFA: -81%. MWA: -75% 12 m: RFA: -86%. MWA: -80%	Significantly decreased at 6 and 12 m. No difference between RFA and MWA	Improved at 6 and 12 m. No difference between RFA and MWA	3-19.9 m
Chorti and cols. (^25^) 2015-2021	RCT: 3 Retrospective: 11 Prospective: 2	3-179 mL	RFA: 1576 MWA: 1284 LA: 501 HIFU: 91 CS: 660	Largest volume reduction ranked: CS, followed by RFA and LA. No significant differences	Improvement. No significant differences among LA, MWA or RFA	Improvement. No significant differences among LA, MWA or RFA	3-60 m
Ha and cols. (^26^) 2002-2012	RCT: 8 Prospective: 2	7.5-13.3 mL	RFA: 65 LA: 119	LA: 49.9 (41.4%-58.5%) RFA: 76.1 (70.1%-82.1%)	NE	NE	6-12 m
Cesareo and cols. (^27^) 2006-2022	RCT: 1 Prospective: 7 Retrospective: 6	1.7-118.1 mL	RFA: 703 LA: 314	NR	RFA: VAS: -3.09 LA: VAS: -2.61	RFA: -1.45 LA: ND	6-24 m
Ji and cols. (^28^) 2011-2020	8 retrospective cohort studies	>13 mL Range NR	RFA: 258 LA: 460 MWA: 215	1 m: 45.3%. 3 m: 65.5%. 6m: 69.1% 12 m: 69.4%	Effectively relieved. SMD: 4.419	Effectively relieved. SMD: 4.245.	3-95.3 m
Xu and cols. (^29^) 2019-2022	5 observational studies	3-65 mL	RFA: 483 LA: 220 MWA: 236	6 m: 61.83%. 12 m: 71.76%. 24 m: 77.98%. 36 m: 78.25%. 48 m: 81.54%. 60 m: 81.43%	NE	NE	5 years
Trimboli and cols. (^30^) 2005-2015	RCT: 3 Prospective: 4 Retrospective: 5	20.1 ± 22.4	RFA: 1186 LA: 2009	RFA 6 m: 68%, 12 m: 75%, 24 m: 87%. LA: 6 m: 48%, 12: 52%, 24 m: 45%, 36 m: 44%	Evaluated only for RFA: SMD: -3.34	Evaluated only for RFA: SMD: -1.45	6-36 m
Cho and cols. (^31^) 2010-2020	Retrospective: 10 Prospective: 2	8.2-36.3 mL	RFA: 695 BTN from 680 patients LA: 528 BTN from 528 patients	RFA 6m: 64.5%, 12m: 76.9%, 24 m: 80.1%, 36 m: 80.3%. LA: 6 m: 48.3%, 12 m: 52.3%, 24 m: 45.5%, 36 m: 45.9%	NE	NE	>3 years
Guan and cols. (^32^) 2014-2019	Cohort study: 6 RCT: 1 Comparison with thyroidectomy	8.6 ± 9.8	MWA: 206 RFA: 345 CS: 561	NE	Improvement. No significant differences between TA and surgery	TA yielded significantly better POP cosmetic effect	6-12 m
Zufry and cols. (^33^) 2017-2023	Retrospective: 7 Prospective: 2	RFA: 67.2 ± 112.5 MWA: 52.2 ± 46.4	RFA: 1332 nodules from 1305 patients. MWA: 1343 nodules from 1276 patients	RFA: 1 m: 35.7%, 3 m: 55.8%, 6 m: 74.4%, 12 m: 83,3. MWA: 1 m: 35.3%, 3 m: 54.3%, 6 m: 69.9%, 12 m: 76.8%	Improvement. No significant differences between RFA and MWA	Improvement. No significant differences between RFA and MWA	6-12 m
He and cols. (^34^) 1998-2015	RCT: 16	27.5 ± 22.1	RFA: 179 EA: 134 LA: 187 HIFU: 21 Control: 322	RFA *vs.* control: 6 m: 79.1%. 12 m: 88.4%. LA *vs.* control: 6 m: 60.8%. 12 m: 79.2%. HIFU *vs.* control: 6 m: 44.9%	Improvement. SUCRA: LA: 69.3, RFA: 56.3	Improvement. SUCRA: LA: 46.9, RFA: 65.4	6-36 m
Ding and cols. (^35^)	Prospective: 4 Retrospective: 9. RCT: 2 NRT: 1	NR	RFA: 664 MWA: 487 HIFUS: 77	SMD -1.77 (significant reduction)	SMD: -1.01 (significant reduction)	SMD: -1.26 (significant Improvement)	6-36 m
Yuan and cols. (^36^)	RCTs: 4 Retrospective: 11 Prospective: 1	2-205 mL	RFA: 1745 MWA: 1432 LA: 812 EA: 66 LIA: 39	SUCRA: RFA group had the highest VRP at 12 months	SUCRA: Greatest improvement with RFA	SUCRA: Greatest improvement with RFA	1-60 m

Abbreviations: VRP: volume reduction percentage; BTN: benign thyroid nodules; RTC: recurrent thyroid cancer; RFA: radiofrequency ablation; MWA: microwave ablation; LA: laser ablation; EA: ethanol ablation; LIA lauromacrogol injection for ablation; CS: conventional surgery; HIFU: high-intensity focus ultrasound; RCT: randomized controlled trial; NRT: nonrandomized trial; m: months; NE: not evaluated; NR: not reported; VAS: Visual Analogic Scale; ND: no data; SMD: standardized mean difference; POP: postoperative; SUCRA: surface under the cumulative ranking curve.

1. Volume reduction percentage (VRP), which calculates the percentage reduction in nodule volume following ablation treatment relative to the baseline volume using the formula [(V0-V1)/V0] × 100, where V0 is the baseline nodule volume and V1 is the post-ablation nodule volume at a specific follow-up interval (^37^);
2. Symptom resolution or improvement, evaluated using the visual analog scale;
3. Resolution of cosmetic concerns, assessed using a specific scale;
4. Nodular regrowth or recurrence, defined as an increase in nodule volume by over 50% compared to the last ultrasonographic examination following image-guided thermal ablation intervention (^37^).

To ensure a comprehensive evaluation, meta-analyses that focused solely on a single technique (*e.g.*, RFA, MWA, or LA) were excluded during the effectiveness assessment in favor of studies that considered various TA techniques. Conversely, in the safety assessment, systematic reviews that addressed only one technique were included due to the scarcity of studies concentrating exclusively on safety. Most meta-analyses that primarily focused on effectiveness also discussed safety as a secondary topic.

The analyzed meta-analyses confirm that the results show a reduction in nodule volume, symptom improvement, and resolution of cosmetic concerns. Nonetheless, it is important to note that not all systematic reviews evaluated all these variables (VRP, symptom, and cosmetic concern).

In terms of nodular regrowth, it was observed that over a 5-year follow-up period, a small percentage of patients experienced regrowth (10.6%), with some requiring a second procedure (9.6%) (^32^). The RFA was found to be the safest intervention in terms of preventing nodular regrowth (^25^,^28^), supported by Cho and cols. (^31^), who documented the long-term maintenance of treatment efficacy and showed RFA to lead to less regrowth and a lower need for delayed surgery than LA.

Some meta-analyses have compared various TA modalities and their outcomes to those of surgery. Despite significant research gaps, primarily due to a lack of long-term data and high-quality RCTs (^25^), the evidence consistently shows the effectiveness of these modalities in terms of VRP, and symptomatic and cosmetic improvement. Guan and cols. (^32^) found no significant difference in symptom improvement between TA and surgery but observed significantly better cosmetic outcomes with TA. Furthermore, specific systematic reviews have compared the efficacy and safety of different TA techniques, identifying RFA as superior in terms of VRP, symptom improvement, and reduced nodular regrowth compared to MWA (^36^) or LA (^33^,^34^), even in meta-analyses that included only RCTs (^26^). Yuan and cols. (^36^) conducted a network meta-analysis confirming RFA as the most efficacious modality in these regards.

The scarcity of RCTs comparing different TA techniques contributes to the low quality of this evidence. Nonetheless, Cesareo and cols. conducted a randomized, parallel, open-label trial demonstrating a significantly greater reduction in nodule volume with RFA compared to LA at six (^38^) and 12 months (^10^).

### Safety

Two systematic reviews assessed safety (^39^,^40^), while five reviews (24,28,30-32) also focused on effectiveness, addressed safety concerns, and were included in the analyses (**Table 2**). Safety was evaluated based on complications observed immediately following the procedure and during the follow-up period. Major complications include events that, if not treated, could threaten the patient’s life, result in substantial morbidity and disability, necessitate hospital admission, or significantly prolong the hospital stay. Minor complications consist of common post-ablation syndrome symptoms such as fever, pain, nausea, and vomiting (^41^).

**Table 2:** Safety of thermal ablation in thyroid nodules

Study/period	Number and type of included studies	Total number of patients	TA employed	Complications		Follow-up, months
				Major	Minor	
Chung and cols. (^39^) 2007-2016	24: 12 retrospective 9 prospective 3 unclear	BTN: 2245 patients and 2540 nodules. RTC: 176 patients and 246 nodules	RFA Overall complication rate: 2.38%	1.35% Malignant: 6.71% Benign: 1.27%	1.03% Malignant: 4.27% Benign: 0.84%	3-61.3
Wang and cols. (^40^) 2008-2016	32: 17 retrospective 7 prospective 4 case report 4 RCT	BTN: 3409 patients and 3753 nodules	RFA	Voice change/hoarseness: 0.5%-4.7% Extremely rare (<5 reported cases): brachial plexus nerve injury, Horner syndrome, nodule rupture, hypothyroidism	Hematoma (0.9%-17%), fever (0.27%-6%). Extremely rare (<5 reported cases): Skin burn, edema/swelling, vomiting	3-78
Guo and cols. (^24^) 2016-2019	5 cohort studies	1768 patients 1894 BTN	RFA: 899 patients with 956 BTN MWA: 869 patients with 938 BTN	RFA (37/899) (: 4.12%. TVC (35/37) (: 94.6% NR (2/37) (: 5.4%. MWA (50/869) (: 5.75%. TVC (44/50) (: 88%. NR (4/50) (: 8%. SNI (2/50) (: 4%)	RFA (40/899) (: 4.45%. 100% (haemorrhage/hematoma). MWA (36/869) (: 4.14%. haemorrhage/hematoma (33/36) (: 91.7%)	3-19.9
Ji and cols. (^28^) 2011-2020	8 retrospective cohort studies	933 patients with 933 BTN	RFA: 258 LA: 460 MWA: 215	Voice change (1.2%), nodule rupture (0.1%)	Mild pain (8.2%), haematoma (1.5%), swelling (0.6%)	3-95.3
Trimboli and cols. (^30^) 2005-2015	RCT: 3 Prospective: 4 Retrospective: 5	3195 patients	RFA: 1186 LA: 2009	Tracheal injury, Massive colliquative necrosis, brachial plexus injury, Nodule rupture with fasciitis (1/3195) (. Recurrent laryngeal nerve injury: 29/3195	Local pain: 18%. Fever: 5.2% Hematoma: 1.5%. Oedema: 38/3195. Cough: 28/3195. Vasovagal reaction: 18/3195. Bleeding: 16/3195	6-36 m
Cho and cols. (^31^) 2010-2020	Retrospective: 10 Prospective: 2	1223 BTN from 1208 patients	RFA: 695 BTN from 680 patients. LA: 528 BTN from 528 patients.	RFA: 1.3% LA: 1.8%	RFA: 3.3% LA: 0.6%	>3 years
Guan and cols. (^32^) 2014-2019	Cohort study: 6 RCT: 1 Comparison with thyroidectomy	1112 patients	MWA: 206 RFA: 345 Surgery: 561	Hoarseness: TA: 1.56% *vs.* CS: 4.78%. Hypothyroidism: TA: 0% *vs.* CS: 31.1%	POP pain: TA: 32.24% *vs.* CS: 62.59%	

Abbreviations: BTN: benign thyroid nodules; RTC: recurrent thyroid cancer; RFA: radiofrequency ablation; RCT: randomized controlled trial; TVC: transient voice change; NR: nodule rupture; SNI: sympathetic nerve injury; CS: conventional surgery; POP: postoperative.

Adverse events are categorized as follows (^2^):

1. Group 1 (major complications): Horner’s syndrome, definitive recurrent laryngeal nerve palsy, compressive hematoma, and subcutaneous abscess.
2. Group 2 (minor complications): transient dysphonia, non-compressive hematoma, and nodule rupture requiring conservative treatment.
3. Group 3 (side effects): pain during or following the procedure, thyroiditis, thyroid dysfunction, and skin burns.

The overall risk of complications is reported to be 2%-3%, with a risk of permanent complication or severe injury below 1%. Most complications can be managed conservatively, without the need for invasive measures (^42^). The primary major complications include voice changes, while ruptured nodules and permanent hypothyroidism are extremely rare (^39^).

Recently, Chorti and cols. (^25^) conducted a systematic review and a Bayesian network meta-analysis, evaluating vocal disturbances, laryngeal nerve palsy, hypothyroidism, and nodular regrowth as secondary endpoints. According to SUCRA rankings, RFA was identified as the intervention least likely to result in vocal complications and thyroid gland dysfunction. This finding was corroborated by another network meta-analysis, which highlighted RFA as the procedure with the lowest probability of complication rates based on SUCRA analyses (^28^).

### Accessibility and costs

The accessibility of TA in Latin American countries has been increasing. Given that meta-analyses have shown TA for BTN may offer potential advantages in terms of safety, cosmetic effects, patient satisfaction, post-operative quality of life, and shorter hospitalization time compared with conventional thyroidectomy (^43^), with no statistical differences in symptom improvement (^32^). This evidence suggests that TA could be considered the primary option for managing BTN over conventional surgery in cases where symptoms or cosmetic concerns are present, for patients who are indicated for surgery but decline it, and for nodules larger than 2 cm with evidence of nodular growth during follow-up. In cases of significant nodular growth (defined as an increase of ≥ 20% in at least two nodule diameters with a minimum increment of 2 mm, or nodule volume increase of > 50%, or the presence of local compression symptoms), repeating the fine needle aspiration is recommended (^44^). Core needle biopsy is not advised in these instances, unless cytology results are indeterminate, or there is suspicion of thyroid lymphoma or anaplastic cancer (^45^).

Economic considerations are crucial when discussing this recommendation, and the cost-effectiveness of LA compared to traditional surgery has not yet been firmly established (^46^). RFA is the modality most extensively studied in this context. Yue and cols. (^47^) conducted a retrospective cohort study in China to assess the quality of life and cost-effectiveness of RFA versus open thyroidectomy. Their findings indicated that patients treated with RFA experienced significantly better health-related quality of life and quality-adjusted life years compared to those treated with open thyroidectomy. In the Chinese healthcare setting, where the cost of RFA is USD 2,740 and open thyroidectomy is USD 1,872, RFA may not be considered cost-effective unless the price of the RFA device is reduced by 30%.

It is worth noting that cost-effectiveness and savings largely depend on the cost of the RFA probe; thus, if the cost per RFA probe can be reduced to less than USD 1,500 in the USA, RFA would represent a considerable cost-saving option for treating thyroid nodules (^48^). Similarly, Kuo and cols. (^49^) used a patient-level state transition microsimulation decision model that found RFA to be a more cost-effective treatment strategy than thyroidectomy for most patients with benign, nonfunctional thyroid nodules. However, this finding is highly sensitive to the cost of RFA (USD 5000.00).

Evidence regarding cost-effectiveness at the Latin American level remains scarce. Nonetheless, Schalch and cols. (^50^) conducted a prospective cost-effectiveness analysis comparing RFA and conventional thyroidectomy, finding that RFA represented 76% of the cost of partial thyroidectomy, demonstrating RFA’s competitive cost and effectiveness as an alternative in treating BTN.

Regarding MWA, a prospective trial compared this TA modality with surgery, indicating that patients undergoing MWA reported better general and mental health scores at 6 and 12 months. The mean total cost was also lower (^51^).

### Comments

Many studies included in the meta-analyses were observational, covering both non-functioning and autonomously functioning BTNs, leading to a high degree of inconsistency and heterogeneity. Consequently, the level of evidence in these reviews ranged from low to moderate. Moreover, RFA is the predominant technique represented. While not all meta-analyses focused directly on safety, data regarding safety measures were analyzed and included as part of a comprehensive examination of this aspect.

One consideration to bear in mind is that some studies suggest TA may be less effective in larger nodules. However, Ji and cols. (^28^) conducted a systematic review on this issue, finding TA to be an effective technique that could serve as an alternative for managing large BTN. Nonetheless, Trimboli and cols. (^30^) analyzed the VRP of BTN based on baseline nodule volume and discovered that nodules smaller than 30 mL achieved better outcomes than larger ones (VRP at 12 months: 75% *vs.* 63%).

It is worth noting that, based on reviews demonstrating the effectiveness, safety, patient preferences, and potential cost savings of using TA for treating BTN compared to surgery, this approach could potentially emerge as the primary therapeutic option. However, given the scarcity of cost-effectiveness research in Latin America, exploring whether TA could serve as the first-line therapeutic choice for BTN, especially when considering economic evaluations, is crucial.

### Autonomously functioning thyroid nodules

AFTN affect 2.5% of adults globally and are typically benign, accounting for 5%-10% of all thyroid nodules (^52^), thus representing the second most common cause of hyperthyroidism (^53^). The incidence of AFTN of 1.6-3.6 cases per 100,000 person-years (^54^). AFTN presents as hyperfunctioning or “hot” nodules in thyroid scintigraphy (^55^). Traditionally, AFTN have been treated with anti-thyroid medications, radioactive iodine (RAI), or surgery, each carrying specific risks. It is crucial to emphasize that definitive treatment is warranted for this condition, as some patients may not be suitable candidates for RAI or surgery, thus leading to prolonged anti-thyroid medications with the risk of adverse effects such as agranulocytosis, vasculitis, or liver failure. RFA has emerged as a non-surgical treatment alternative for AFTN, exhibiting excellent efficacy and a minimal risk of complications (^56^,^57^). Some reviews have indicated that RFA, the most extensively studied treatment modality for TA, has demonstrated a VRP ranging between 69.4%-76.4% at 12 months, with 50%-90.9% of patients achieving normalization of thyroid function (^58^). Another literature review by Cesareo and cols. (^59^) on RFA included 6 prospective and 2 retrospective studies (205 patients), with a mean initial volume between 5-27.7 mL. The follow-up duration ranged from 6-24 months, with a VRP between 50.7%-86%, and thyroid function normalization of 24%-86%.

The most recent narrative reviews, conducted by Podrat and cols. (^56^) and Muhammad and cols. (^53^), indicated a variable VRP of 32.45%-86.1%, as well as TSH normalization rates of 21.7%-100%. The effectiveness of RFA for AFTN varies widely. Upon analyzing the reasons for this variability, it is noteworthy that the more recent studies showing greater efficacy, potentially due to advancements in technique, such as the fixed electrode technique, which is no longer the current accepted technique (i.e., the moving shot technique). This underscores the potential impact of the technique employed on achieving biochemical control, emphasizing the importance of technology and evolving technique knowledge on improved symptom control and VRP.

Several medical societies have published recommendations for identifying candidates with AFTN for TA (8,12-19), addressing the successful use of ablative techniques for AFTN causing subclinical or overt hyperthyroidism. Notably, large AFTN (>20 mL) may be less responsive (^13^,^14^), leading European guidelines to advise against using TA as a first-line treatment for large AFTN and to consider them only for young patients with small AFTN (^8^). Other guidelines suggest TA as a safe alternative if first-line treatments (surgery/RAI) are contraindicated (12,15,16,18,21).

### Literature research

A search of the MEDLINE database was conducted to identify original studies reporting the efficacy and safety of thermal ablation in patients with benign non-functioning thyroid nodules. The search terms used were: (((“laser ablation”) OR (“radiofrequency ablation”) OR (“microwave ablation”) OR (“thermal ablation”)) AND ((“autonomously functioning thyroid nodule”) OR (“hyperthyroidism”) OR (“AFTN”))). The literature search was not confined to a specific date range and was updated until April 1, 2024.

### Inclusion criteria

Studies were eligible for inclusion if they were meta-analyses or systematic reviews of RCTs or observational studies involving patients with AFTN treated with thermal ablation, and they contained follow-up results related to effectiveness or safety.

### Effectiveness

Three systematic reviews were included to evaluate effectiveness (**Table 3**) (^60^-^62^). The primary metrics for assessment were VRP and TSH normalization. Radiofrequency ablation is the most thoroughly researched treatment for TA, followed by LA. There is limited data on MWA, with only one retrospective study involving 30 patients that compared MWA with RAI. This study showed no significant difference in nodule volume reduction or therapeutic success as gauged by TSH and free thyroxine (fT4) levels, coupled with a lower risk of post-treatment hypothyroidism (^63^).

**Table 3:** Effectiveness of thermal ablation in autonomously functioning thyroid nodules

Study/period	Number and type of studies	Initial nodular volume	TA employed. # patients	Effectiveness/improvement		Follow-up
				VRP	TSH normalization	
Kim and cols. (^60^) 2007-2019	Retrospective: 9 Prospective: 5	7.2-55.3	LA: 82 RFA: 244	Last follow-up: 44 to 86.6%	71.2%. >18 mL: 67% ≤18 mL: 73.6% LA: 74.8%. RFA: 69.9%	Mean: 12.8 m
Cesareo and cols. (^61^) 2008-2018	Retrospective: 2 Prospective: 6	18.5 ± 30.1	RFA: 205	6 m: 52.1–86.1%. 12 m: 74.7%-86.1%	57% (21.7%-87.5%)	6–24 m
Giovanella and cols. (^62^) 1984-2023	Retrospective: 14 Prospective: 6 RCT: 1 NS: 2	NR	RFA: 296 RAI:1042	45.8%-74.8%	12 months: Success rate: RAI: 93% RFA: 63%	6 m–6.2 ± 2.9 years

Abbreviations: VRP: volume reduction percentage; RFA: radiofrequency ablation; LA: laser ablation; RAI: radioactive iodine; NS: not specified; NR: not reported; RCT: randomized controlled trial; m: months; NE: not evaluated.

Studies comparing RFA with other therapeutic options are scarce. RFA was significantly less effective than surgery in normalizing thyroid function (^59^), and patient satisfaction with symptom resolution did not align with that following surgery (^64^). Cervelli and cols. (^65^) found no significant difference in post-treatment nodule volume reduction and hyperthyroidism resolution when comparing RFA with RAI.

A meta-analysis by Giovanella and cols. (^62^) revealed RAI’s superiority over TA in terms of success rates. Nonetheless, a nuanced analysis of this data reveals that only three studies drew direct comparisons, showing moderate heterogeneity and a non-significant risk of treatment failure when comparing TA with RAI (RR, 1.24; 95% CI, 0.94-1.63, *p* = 0.12). Specifically, in the three studies that directly compared TA with RAI, only one evaluated RFA without finding a statistical difference between the post-treatment reduction in nodule volume and the resolution of hyperthyroidism (^65^). Similarly, the study that evaluated MWA did not show any statistically significant difference in therapeutic success (^63^). The third study (^66^), published in 2007, utilized LA and was the only one inferior to RAI. On the contrary, twenty-seven reports were included for indirect comparative analysis, revealing high heterogeneity associated with the indirectness of the results. Unlike direct comparisons, the indirect analysis showed the superiority of RAI, albeit with a very low level of evidence, given the issues of inconsistency and indirectness in the evidence.

### Safety

No systematic reviews solely assessing this criterion were found; however, one meta-analysis that evaluated effectiveness also addressed safety concerns (**Table 4**) (^60^). Comparisons between RFA and surgical resection demonstrated lower complication rates, preserved thyroid function, and reduced hospitalization times (^67^). Additionally, compared to RAI, RFA does not involve radiation exposure and poses a lower risk of post-treatment hypothyroidism (^65^). The low complication rate, with self-limited voice changes and no reported cases of post-procedure hypothyroidism or hypocalcemia, underscores the safety of thermal ablation techniques compared to RAI and surgery (^56^).

**Table 4:** Safety of thermal ablation in autonomously functioning thyroid nodules

Study/period	Number and type of included studies	Total number of patients	TA employed	Complications		Follow-up, months
				Major	Minor	
Kim and cols. (^60^) 2007-2019	Retrospective: 9 Prospective: 5	326	LA: 82 RFA: 244	1 patient with voice change that improved within 1 month	0	Mean: 12.8 m

Abbreviations: RFA: radiofrequency ablation; LA: laser ablation.

### Accessibility and costs

As previously mentioned, these therapeutic options have become increasingly affordable and accessible for treating BTN. While evidence regarding AFTN is growing, direct comparisons in terms of economic evaluations, including cost-effectiveness between TA and RAI, are lacking. In fact, the cost-effectiveness of LA compared to RAI has yet to be firmly established (^46^). This lack of comparative data makes recommending these techniques as first-line therapeutic options difficult. It is possible to extrapolate cost-effective data from surgery for BTN, although this cannot be asserted for RAI. Given this evidence, we could consider TA, especially RFA, the most studied modality for AFTN, as a suitable option for treating AFTN when surgery or RAI are contraindicated.

### Comments

The volume has been considered a key factor to predict success in AFTN. For instance, some societies consider that large AFTN (>20 mL) may exhibit lower responsiveness (^13^,^14^). Mauri and cols. (^68^) found a significant difference in the rate of patients who discontinued medical therapy at 12 months based on the baseline volume (<10 mL: 74% *vs.* >30 mL: 19%). Furthermore, technical variables, such as delivering > 2.1 kJ/mL during RFA, have been demonstrated to predict cure in AFTN (91% *vs.* 44%) (^69^), along with nodular vascularity on ultrasound for normalization in TSH and volume reduction (^70^).

RFA is the most researched TA modality in the field of AFTN. The reported effectiveness varies significantly, probably due to the initial size of the nodule and the technical methods employed. The safety profile of TA is high, surpassing that of surgery or RAI, especially when achieving a euthyroid state without the need for thyroid hormone replacement. Despite the promising effectiveness of RFA, the lack of RCTs comparing TA to RAI or surgery for AFTN results in a low level of evidence. Additionally, there is insufficient data on regrowth and recurrence of hyperthyroidism during long-term follow-up.

Recently, a group of specialists in thermal ablation therapies from Latin America published their experiences in managing AFTN. This retrospective, observational, multicenter cohort study included patients with a solitary AFTN that was histologically confirmed as benign and treated with a single session of RFA. A total of 81 patients with a solitary, benign AFTN were enrolled. The VRP consistently increased over the follow-up period, with median reductions of -50%, -74.9%, -78.4%, and -90.2% at 1, 3, 6, and 12 months, respectively. The rate of hyperthyroidism resolution was 93.8% (76/81). Following the RFA, 58.02% of patients (47/81) normalized their thyrotropin levels within 1 month of follow-up, and by 3 months, an additional 33.3% achieved normalization (27/81). Notably, baseline volume ≥ 10, 20, or 30 mL) did not affect the achievement of clinical success. In bivariate analyses, a VRP ≥ 50% at the 6-month follow-up was associated with the resolution of hyperthyroidism (^71^).

Overall, complications occurred in 6.2% of patients (5/81), including a major complication (of transient Horner syndrome in 1.2% (1/81), transient dysphonia in 3.7% of cases 3/81), and hypothyroidism requiring low-dose levothyroxine replacement in 1.2% (1/81). In conclusion, the results of this multicenter study suggest that RFA is a promising treatment option for patients with solitary AFTN, regardless of their baseline characteristics, including volume, age, or composition. Further research is crucial to establish TA as an alternative for the management of AFTN, to assess its long-term results, and to evaluate its cost-effectiveness compared to RAI or surgery.

### Papillary thyroid microcarcinoma

The management of thyroid cancer has evolved from traditional treatments such as surgery and RAI to the American Thyroid Association guidelines’ recommendation of active surveillance (AS) for selected low-risk PTMC (^72^,^73^). PTMC is recognized as the most common neoplasm, raising concerns about overdiagnosis and overtreatment, especially in cases categorized as low-risk, which are typically associated with a favorable prognosis (^74^).

The AS approach may cause anxiety in some patients as the tumor is left untreated (^4^). In contrast, others may not be suitable candidates due to the need for repeated and regular follow-up ultrasound examinations and appointments. In such cases, TA modalities may offer a viable therapeutic alternative (^75^), especially considering that they provide patients with a minimally invasive curative option compared to surgical resection or AS, and may offer an option that can be preferred by some patients (^76^).

Despite the increasing evidence demonstrating its effectiveness and safety in these patients (^77^), several international societies that have addressed TA recommendations have given limited attention to its application for primary PTMC management (^78^). For instance, some societies recommend TA for patients at high surgical risk or those who decline surgery (^13^), while others suggest that in cases of low-risk PTMC where AS is an option, RFA may also be considered as an alternative (^15^). Additionally, it is not recommended as a first-line treatment but rather as an option for PTMC in cases where it is unifocal, shows no metastatic lymphadenopathy, and the patient is at high risk, does not accept the AS, or refuses surgery (^18^). However, as mentioned earlier, many consensus statements do not cover this topic (8,12,14,16). Furthermore, the most recent American Thyroid Association statement only addresses BTN (^17^).

The current recommendations for using TA in primary thyroid carcinoma apply to low-risk papillary thyroid cancer (PTC) patients who meet the criteria for AS but decline surgery or AS, including those with either PTMC (one cm or smaller, T1a) or PTC smaller than two cm (^72^). Nonetheless, several factors need to be taken into account before considering this alternative approach, such as multifocal PTMC and tumor location, among others. Molecular genetic testing has emerged as a valuable tool for diagnosis and treatment (^79^,^80^). However, there are no clear guidelines regarding its assessment before TA. It is worth noting that a positive test for the BRAF V600E mutation has not been linked to local tumor progression after RFA (^81^) and is considered an effective and safe therapeutic option in these patients (^82^). Conversely, a molecular profile showing the presence of TERT promoter and TP53 mutations, indicating a high-risk molecular profile (^83^), serves as a contraindication for employing TA (^72^).

The presence of high-risk features such as multifocality, capsular invasion (^84^,^85^), subcapsular tumor location with a distance of ≤ 2 mm from capsule or trachea (^81^), lymph node metastasis (LNM), tumor enlargement (maximal diameter increase ≥ 3 mm), and extrathyroidal extension (^86^) necessitates surgery as the primary treatment option for these patients. However, in cases where surgery is not feasible due to patient-related conditions (*e.g.*, severe associated comorbidities) or tumor behavior, TA or AS could be considered based on the patients’ preferences, tumor features, available medical resources, and other relevant factors (^74^).

### Literature research

A comprehensive search was conducted in the MEDLINE database to identify original studies that report on the efficacy and safety of thermal ablation in patients with benign non-functioning thyroid nodules. The search strategy employed the following terms: ((“laser ablation”) OR (“high-focused ultrasound”) OR (radiofrequency) OR (RFA) OR (laser) OR (laser ablation) OR (thermal ablation) OR (“radiofrequency ablation”) OR (“microwave ablation”) OR (“thermal ablation”)) AND ((“papillary thyroid cancer”) OR (“papillary thyroid carcinoma”) OR (“papillary thyroid microcarcinoma”)). The literature search was conducted without a specific date limit and was updated until April 1, 2024.

### Inclusion criteria

Studies were included if they met the following criteria: (a) meta-analyses or systematic reviews of RCTs or observational studies involving patients with PTC treated with thermal ablation, and (b) contained follow-up results relating to effectiveness or safety.

### Effectiveness

Twenty-two systematic reviews were evaluated for effectiveness, with 14 ultimately included. The variables considered in this assessment included:

1. Volume reduction percentage;
2. Local recurrence, defined as the recurrence of PTC at the original ablation site or the identification of new PTC lesions within the thyroid;
3. Lymph node metastasis;
4. Comparison with surgery, encompassing ope­ration time and hospital stay duration.

To ensure a thorough evaluation that encompasses the various TA techniques, meta-analyses that only focused on a single technique were excluded from the effectiveness assessment, similar to the approach taken with non-functioning BTNs. The included meta-analyses demonstrated that TA effectively reduces nodule volume, ensures a high rate of complete tumor disappearance, and maintains low rates of local recurrence, lymph node metastasis (LNM), and distant metastasis, thereby diminishing the necessity for surgical intervention during follow-up. Notably, several meta-analyses that compared TA with surgery found no statistically significant differences in recurrence rates, LNM, or the requirement for salvage or delayed surgery during follow-up (^87^-^91^) (**Table 5**).

**Table 5:** Effectiveness of thermal ablation in papillary thyroid carcinoma

Study/period	Number and type of studies	Population/comparator	TA employed. # patients	Effectiveness		Follow-up
				CDP/VRP	Recurrence/LNM	
Li and cols. (^87^) 2017-2022	Retrospective: 29 All conducted in China	PTMC/Among TA modalities or surgery*	MWA: 1455 LA: 70 RFA: 1017	NE	No statistical differences. The maximum SUCRA was 64.4% for MWA (lowest likelihood of recurrence). LNM: 67.8% for RFA (lowest likelihood of LNM)	NS
Gao and cols. (^104^) 2018-2022	Retrospective: 35 Prospective: 1	PTC T1N0M0/TA modalities	RFA: 2475 MWA: 1495 LA: 404	VRP: RFA: 99.89%, MWA: 99.19%, LA: 100% CDP: RFA: 81%, MWA: 71%, LA: 93%	LR: RFA: 2%, MWA: 2%, LA: 3%. LNM: RFA: 1%, MWA: 2%, LA: 2%	6.45 ± 4.92 to 111.6 ± 20.7 m
Chen and cols. (^95^) 2011-2022	Meta-analysis: 1 Retrospective: 23 Case: 1	PTMC/none	RFA: 2120 MWA: 1023 LA: 131	VRP: 64.9%-100% RFA: 98.52% MWA: 94.79% LA: 92.98% CDP: NE	Recurrence: 1.4% LNM: 0.73%	11-72 ± 18 m
Zhang and cols. (^94^) 2021-2023	Retrospective: 4	MPTMC/none	RFA: 47 p and 100 n. MWA: 110 p and 263 n. LA: 12 p and 26 n.	VRP: RFA: 99.94, LA: 100%, MWA: 100%. CDP: RFA: 92% MWA: 79.5%, LA: 100%	LR: 1.18%. LNM: 1.78%. During the follow-up, neither distant metastasis nor the need for surgery was observed	18-60 m
Ledesma and cols. (^88^)	Transversal: 2 Prospective: 5 Retrospective: 6	Low-risk PTC ≤ 1.5 cm/surgery	Surgery: 1983. AS: 1495. MWA: 255. LA: 36. RFA: 265	NE	Recurrence: No difference between surgery and TA or AS	5.5-64.2
Wang and cols. (^105^)	Retrospective: 10 Prospective: 1	PTC T1N0M0/TA modalities	T1aN0M0: RFA: 131 LA: 139 MWA: 576. T1bN0M0: RFA: 369	MD of VRP: MWA 24 m: 98.34% RFA 24 m: 93.69%	Disease progression (recurrence and LNM): MWA: 1%, RFA: 3.1%	22.8-65.4 ± 6.3
Cho and cols. (^99^) 2010-2017	Retrospective: 7 Prospective: 2	PTMC/TA modalities	LA: 101 RFA: 102 MWA: 267	VRP: LA: 100%, MWA: 92.2%, RFA: 82.6%. CDP: LA: 98.65%, MWA: 61.1%, RFA: 61.2%	LR: 0% LNM: LA: 1.98%, MWA: 0%, RFA: 0%	NS
Tong and cols. (^107^) 2005-2018	Retrospective: 9 Prospective: 3	PTMC/Surgery or TA modalities	LA: 101 MWA: 778 RFA: 697 Surgery: 143 (only one study)	VRP: No significant differences. SMD: MWA: -3.82, RFA: -1.35, LA: -1.8. CDP: No significant differences. RFA: 76.2%, MWA: 62.9%, LA: 57.3%	LR: No significant differences. RFA: 0.01%, MWA: 0.85%, LA: 1.87% LNM: RFA: 0.6%, MWA: 1.3%, LA: 2%. No distant metastasis occurred	3-65
Kim and cols. (^89^) 2012-2013	Retrospective: 4	Low-risk PTMC/surgery	MWA: 209 RFA: 94 LA: 36 Surgery: 314	NE	No LR or distant metastasis were observed. New tumor: surgery group: 1.3%, TA: 1.4%. LNM: Surgery group: 3.3%, TA: 2.6%. Rescue/delayed surgery: Surgery group: 1.6%, TA: 2.6%. No significant differences	25.1-64.2 m
Xue and cols. (^93^) 2018-2022	Retrospective: 13	PTC/TA modalities	MWA: 1641 RFA: 926	VRP at 3 years: MWA: 99.9%, RFA: 98.78%. 98.91% CDP at 3 years: MWA: 70%. RFA: 94%**	Newly discovered PTC at 3 years: 0.3%. LNM: 0%	At least 3-year follow-up
Cho and cols. (^92^) 2008–2017	Retrospective: 3	PTMC/TA modalities	RFA: 166 MWA: 41	VRP: RFA: 100%, MWA: 99.4% Disappearance: RFA: 100%, MWA: 97.6%	LR: 0% No distant metastasis Surgery requirement during follow-up: 0% New cancer: RFA: 2.4%. MWA: 0%	At least 5-year follow-up
Shen and cols. (^90^) 2018-2020	Retrospective: 7	PTMC/surgery	RFA: 94 LA: 36 MWA: 312 Surgery: 425	NE	Recurrence: No significant differences. Shorter hospital stay**	NS
Hurtado and cols. (^91^) 2018-2023	Retrospective: 8 Prospective: 1 Clinical trial: 1	PTMC/surgery	RFA: 426 LA: 36 MWA: 1006 Surgery: 1471	NE	LR: No significant differences. LNM: No significant differences.	1–64 m
Choi and cols. (^108^) 2010-2018	Retrospective: 7 Prospective: 2 Unclear: 2	PTMC	MWA: 389 RFA: 195 LA: 131	VRP: MWA: 95.3%, RFA: 99.3%, LA: 88.6% CDP: MWA: 56.5%, RFA: 65.2%, LA: 48.7%	Recurrence: 0.4% LNM: NE	3-60 m

Abbreviations: LNM: lymph node metastasis; MWA: microwave ablation; RFA: radiofrequency ablation; LA: laser ablation; AS: active surveillance; TA: thermal ablation; NS: not specified; NR: not reported; VRP: volume reduction percentage; CDP: complete disappearance percentage; RCT: randomized controlled trial; m: months; P: patients; N: nodules; NE: not evaluated; PTMC: papillary thyroid microcarcinoma; SMD: standardized mean difference; MD: mean difference; PTC: papillary thyroid cancer; MPTMC: multifocal papillary thyroid microcarcinoma; SUCRA: surface under the cumulative ranking curve; LR: local recurrence.

* Total thyroidectomy or lobectomy (n = 2247 patients).

** Statistically significant.

When comparing different TA modalities, no statistically significant differences in recurrence or LNM were found. Nonetheless, SUCRA analyses indicated that MWA exhibited the lowest recurrence risk, and RFA the lowest LNM (^87^) risk. The findings regarding VRP or complete disappearance percentage (CDP) were variable, with LA being underrepresented due to fewer patients and less inclusion in meta-analyses that evaluated long-term follow-up (^91^,^92^). The analyzed studies show that RFA and MWA were the most well-represented techniques, displaying comparable effectiveness in VRP or CDP. Yet, RFA demonstrated a significantly higher CDP at the 3-year follow-up (^92^). The patient population in these meta-analyses generally consisted of individuals with PTMC or T1 (2 cm or less), exhibiting solitary lesions without evidence of extrathyroidal invasion, lymph node, or distant metastasis (^87^). The management of multifocal PTMC requires particular consideration. A recent meta-analysis assessed the effectiveness of TA in treating multifocal PTMC. The inclusion criteria for this analysis were analogous to those previously mentioned, with the addition of patients deemed unsuitable for surgery or those who declined it (^94^).

### Safety

No systematic reviews have been conducted solely on safety assessment; however, the majority of the meta-analyses included also addressed safety. Consequently, 13 studies were analyzed, showing (**Table 6**). TA demonstrated significantly fewer complications compared to surgery (^89^,^90^), as well as reduced intra-operative blood loss (^87^). The comparison of different TA modalities revealed RFA as the safest technique (^86^,^87^,^95^), with the lowest rates of major and minor complications compared to MWA or LA, confirmed by SUCRA analyses (^87^).

**Table 6:** Safety of thermal ablation in papillary thyroid carcinoma

Study/period	Number and type of studies	Population/comparator	TA employed. # patients	Complications		Follow-up, months
				Major	Minor	
Li and cols. (^87^) 2017-2022	Retrospective: 29 All conducted in China	PTMC/Among TA modalities or surgery*	MWA: 1455 LA: 70 RFA: 1017	The percentage of complications was not specified. The maximum SUCRA was 88.8% for RFA (lowest risk of total complications). TA resulted in significantly fewer complications compared to surgery		
Gao and cols. (^104^) 2018-2022	Retrospective: 35 Prospective: 1	PTC T1N0M0/none	RFA: 2475 MWA: 1495 LA: 404	Not available Data (too low)	RFA: 3% MWA: 13% LA: 6%	6.45 ± 4.92 to 111.6 ± 20.7 m
Chen and cols. (^95^) 2011-2022	Meta-analysis: 1 Retrospective: 23 Case: 1	PTMC/none	RFA: 2120 MWA: 1023 LA: 131	Mainly minor. Did not discriminate. RFA: 7.66% LA: 33.13% MWA: 20.02%		6-32 m
Zhang and cols. (^94^) 2021-2023	Retrospective: 4	MPTMC/none	RFA: 47 p and 100 n. MWA: 110 p and 263 n. LA: 12 p and 26 n.	1.78% (transient hoarseness)	3.55% (local pain, bleeding)	18-60 m
Ledesma and cols. (^88^)	Transversal: 2 Prospective: 5 Retrospective: 6	Low-risk PTC ≤ 1.5 cm/surgery	Surgery: 1983. AS: 1495. MWA: 255. LA: 36. RFA: 265	2.27% MWA: 10 (9 TVC and 1 persistent voice change) RFA and LA: 0	0.46% MWA: 1 (cough) LA: 1 (hyperthyroidism) RFA: 0	5.5-64.2
Wang and cols. (^105^)	Retrospective: 10 Prospective: 1	PTC T1N0M0/TA modalities	T1aN0M0: RFA: 131 LA: 139 MWA: 576. T1bN0M0: RFA: 369	MWA: 6.3% RFA: 4.1%		22.8-65.4 ± 6.3
Cho and cols. (^99^) 2010-2017	Retrospective: 7 Prospective: 2	PTMC	LA: 101 RFA: 102 MWA: 267	MWA: 4.12% (TVC) LA: 0.99% (TH) RFA: 0	MWA: 0.75% (bleeding). LA: 0.99% (pain) RFA: 0	NS
Tong and cols. (^107^) 2005-2018	Retrospective: 9 Prospective: 3	PTMC/Surgery or TA modalities	LA: 101 MWA: 778 RFA: 697 Surgery: 143 (only one study)	RFA: 2.7% (TVC) MWA: 4.4% (TVC) LA: 1% (Hypothyroidism)	RFA: 1.3% (skin burn, pain, bleeding). MWA: 2.8% (bleeding). LA: 1% (pain)	3-65
Kim and cols. (^89^) 2012-2013	Retrospective: 4	Low-risk PTMC/surgery	MWA: 209 RFA: 94 LA: 36 Surgery: 314	0%	Surgery: 7.8% RFA: 0%, MWA: 4.2%, LA: 2.2%. Significantly higher in the surgery group (p = 0.03)	25.1-64.2 m
Cho and cols. (^93^) 2008-2017	Retrospective: 3	PTMC	RFA: 166 MWA: 41	RFA: 0.6% (1 patient)-voice change. MWA: 2.44% (voice change)	RFA: 1.8% MWA: 2.44% Hematomas, first-degree burn	At least 5-year follow-up
Shen and cols. (^90^) 2018-2020	Retrospective: 7	PTMC/surgery	RFA: 94 LA: 36 MWA: 312 Surgery: 425	Patients undergoing TA had a lower risk of complication after the treatment (OR 0.24, 95% CI 0.13 to 0.43, p < 0.001)		NS
Hurtado and cols. (^91^) 2018-2023	Retrospective: 8 Prospective: 1 Clinical trial: 1	PTMC/surgery	RFA: 426 LA: 36 MWA: 1006 Surgery: 1471	Hoarseness: RR: 0.29 for TA *vs.* surgery	Infection and hematoma, no statistically significant differences	1-64 m
Choi and cols. (^108^) 2010-2018	Retrospective: 7 Prospective: 2 Unclear: 2	PTMC	MWA: 389 RFA: 195 LA: 131	RFA and LA: 0% MWA: 2.5%. 10 TVC and 1 PVC	RFA: 2.6%, LA: 0.6%, MWA: 2.6%. Moderate pain, hematoma	3-60 m

Abbreviations: RFA: LNM: lymph node metastasis; MWA: microwave ablation; RFA: radiofrequency ablation; LA: laser ablation; TVC: transient voice change; PVC: permanent voice change; TH: transient hypothyroidism; NS: not specified; NR: not reported; RCT: randomized controlled trial; m: months; NE: not evaluated; PTMC: papillary thyroid microcarcinoma; SUCRA: surface under the cumulative ranking curve.

* Total thyroidectomy or lobectomy (n = 2247 patients).

### Accessibility and costs

Minimally invasive techniques for managing conditions such as low-risk PTC are gaining global acceptance. Nevertheless, there is a lack of studies comparing the cost-effectiveness of TA with AS, a widely used strategy. It is important to note that some patients may not have access to comply with the strict follow-up protocol required for AS. Therefore, TA techniques could offer an opportunity to improve treatment for these patients.

Evidence from several included meta-analyses indicates is that TA modalities are as effective as surgery, with a superior safety profile that may provide potential advantages, particularly in terms of shorter hospital stay (^87^), leading to reduced hospitalization costs (^96^).

Shen and cols. (^90^) demonstrated that patients undergoing TA incurred significantly lower treatment costs compared to the surgical group. Similarly, a systematic review and meta-analysis investigating the economic benefits of TA for patients with PTMC revealed shorter hospitalization and operative times, as well as a reduction in postoperative complications compared to surgery. In terms of cost, TA showed superior economic benefits over surgical treatment for patients with PTMC (^97^). Zhang and cols. (^98^) found that RFA did not differ significantly from surgery regarding effectiveness, while it was associated with a higher quality of life and lower overall costs.

Cho and cols. (^99^). conducted a systematic review that revealed a substantial proportion of patients undergoing AS who ultimately opted for surgery (8.7%-32%), not due to tumor progression or LNM but primarily due to significant anxiety. An age-stratified comparison between AS and RFA indicated that RFA might have a more significant therapeutic impact in younger patients (^100^). Furthermore, certain cost-effective studies focusing on surgery have suggested that surgery could offer long-term economic benefits over AS for younger patients (^101^). It is crucial to further investigate TA as a cost-effective therapeutic option compared to AS, particularly in light of studies confirming the cost-effectiveness of surgery.

### Comments

The development of TA as an alternative for patients with low-risk PTC accentuates the need for consensus statements to identify optimal candidates for these treatments. Interestingly, the latest position statement from the Brazilian Society of Endocrinology and Metabolism does not recognize TA as a treatment option for low-risk PTMC (^102^). The evidence reviewed is of low-level quality, primarily based on retrospective studies from Asian countries, highlighting the need for local research. While LA is minimally represented in treating PTC, RFA has demonstrated high effectiveness and a superior safety profile. TA stands as an alternative for low-risk PTC in patients contraindicated for surgery, unwilling to undergo AS or surgery, and in the absence of comparative studies between AS and TA. This gap is partly because, despite similar tumor characteristics warranting either approach, the ideal candidates for these procedures tend to have high surgical risks or a preference against surgery or AS (^103^,^106^).

In conclusion, TA could be the primary treatment option for non-functioning benign thyroid nodules with symptoms or cosmetic concerns, for patients who decline surgery, and for nodules larger than 2 cm showing growth during follow-up. In the case of multinodular goiter, each nodule should be assessed individually. These recommendations are supported by very low to moderate quality evidence, mainly derived from observational studies and, to a lesser extent, RCTs. Similarly, RFA, the most studied modality for AFTN, could be considered suitable for treating AFTN when surgery or RAI are contraindicated, or when patients decline these options, supported by evidence of very low to low quality. Regarding PTMC, TA could be a viable option for patients at high surgical risk or those who decline surgery. Additionally, for low-risk PTMC where AS is feasible, TA may serve as an alternative, especially for younger patients or those unwilling to undergo AS.

## Data Availability

datasets related to this article will be available upon request to the corresponding author.
